# Detoxifying the heavy metals: a multipronged study of tolerance strategies against heavy metals toxicity in plants

**DOI:** 10.3389/fpls.2023.1154571

**Published:** 2023-05-12

**Authors:** Ujala Ejaz, Shujaul Mulk Khan, Noreen Khalid, Zeeshan Ahmad, Sadia Jehangir, Zarrin Fatima Rizvi, Linda Heejung Lho, Heesup Han, António Raposo

**Affiliations:** ^1^ Department of Plant Sciences, Quaid-i-Azam University, Islamabad, Pakistan; ^2^ Member Pakistan Academy of Sciences, Islamabad, Pakistan; ^3^ Department of Botany, Government College Women University, Sialkot, Pakistan; ^4^ College of Business, Division of Tourism and Hotel Management, Cheongju University, Cheongju-si, Chungcheongbuk-do, Republic of Korea; ^5^ College of Hospitality and Tourism Management, Sejong University, Seoul, Republic of Korea; ^6^ CBIOS (Research Center for Biosciences and Health Technologies), Universidade Lusófona de Humanidades e Tecnologias, Lisboa, Portugal

**Keywords:** heavy metal stress, plant defense mechanisms, genomics and transcriptomics, cell signaling pathways, plant structural and functional biology

## Abstract

Heavy metal concentrations exceeding permissible limits threaten human life, plant life, and all other life forms. Different natural and anthropogenic activities emit toxic heavy metals in the soil, air, and water. Plants consume toxic heavy metals from their roots and foliar part inside the plant. Heavy metals may interfere with various aspects of the plants, such as biochemistry, bio-molecules, and physiological processes, which usually translate into morphological and anatomical changes. They use various strategies to deal with the toxic effects of heavy metal contamination. Some of these strategies include restricting heavy metals to the cell wall, vascular sequestration, and synthesis of various biochemical compounds, such as phyto-chelators and organic acids, to bind the free moving heavy metal ions so that the toxic effects are minimized. This review focuses on several aspects of genetics, molecular, and cell signaling levels, which integrate to produce a coordinated response to heavy metal toxicity and interpret the exact strategies behind the tolerance of heavy metals stress. It is suggested that various aspects of some model plant species must be thoroughly studied to comprehend the approaches of heavy metal tolerance to put that knowledge into practical use.

## 1 Introduction

Anthropogenic and natural activities have resulted in the vast concentration of heavy metals in the atmosphere, which affects humans and other living organisms (Rathoure, 2020; Khalid et al., 2021; Yan et al., 2022). Rapid industrialization, intensive mining processes, and extensive agricultural activities plays crucial role in contaminating the environment with heavy metals (Pant and Tripathi, 2014). Heavy metals polluted soils and water bodies have far ranging effects on various aspects of plant life (Parmar et al., 2013), which include but are not only limited to morphology (Chatterjee and Chatterjee, 2000; Smeets et al., 2013), anatomy (Liza et al., 2020; El-Shabasy, 2021), physiology (Asai et al.), and cell signaling (Calderini et al., 1998; Bartels and Sunkar, 2005). Several plants can cope with high levels of heavy metals, which are generally termed as tolerant species, for example, *Erigeron Canadensis, Arundo donax* L, *Populus alba and Morus alba* (Ahmad et al., 2019b) *Sporobolus diander, Cynodon dactylon, Brachiaria mutica, Digitaria ischaemum, Digitaria longiflora, Eragrostis cynosuroides, Launaea asplenifolia, Stylosanthes scabra* and *Parthenium hysterophorus* (Gautam and Agrawal, 2019) can grow, survive, and exhibit greater tolerance to heavy metals but some species show detrimental symptoms of heavy toxicity, like *Malvestrum coromandelianum, Alternanthera paronychioides, Cyperus rotundus, Ambrosia chamissonis, Hyptis suaveolens Xanthium strumarium* and *Portulacca olearcea* (Gautam and Agrawal, 2019). Many studies are available on the impacts of heavy metals toxicity on plants, and various strategies that were used to overcome the negative impacts of this toxicity such as using biochar can reduce the bioavailability of heavy metal ions in soil environments as well as retain, stabilize, and inactivate toxic heavy metals. (Wang et al., 2020; Mansoor et al., 2021; Yang et al., 2022d; Elkhlifi et al., 2023). Another such example of using chelating agents like EDDS, EDTA, organic and synthetic chelators in soil to reduce the toxic effect of heavy metals in plants (Chen et al., 2020; Gluhar et al., 2020; Yang et al., 2021; Ejaz et al., 2022; Gavrilescu, 2022). There are several review papers available on this subject, most of them focused on one or two aspects of the plants but this review provides deep insight on various aspects from structural and functional biology to genetics, molecular, and cell signaling levels, which integrate to produce a coordinated response to heavy metal stress. It is necessary to study various aspects of plant life under heavy metals stress to decode the exact mechanism of the tolerance in plants. This review provides suggestions for future research on the subject as well as the practical uses of the knowledge we have obtained thus far.

The word heavy metal is usually controversial, and various authors have tried to define it by referencing the density of the metals that are involved (Duffus, 2002). We will define heavy metals in this paper based on their density, which ranges between 5.0g/cm^3^ and atomic weight above 23 units for convenience (Koller and Saleh, 2018). Some heavy metals are fundamental components of biological systems, but most are toxic in higher concentrations (Asati et al., 2016). Some heavy metals are required, within permissible limits only, for plant growth, and these types of elements are called essential heavy metals. These include Zinc (Zn), Iron (Fe), Manganese (Mn), Cupper (Cu), Nickel (Ni) (Asati et al., 2016; Anum et al., 2019). The threshold range of heavy metals in plants, below which they are beneficial and above which they become toxic, varies depending on the specific heavy metal and the plant species. Zn is an essential micronutrient for plants and is beneficial for growth and development at low concentrations. However, excessive uptake of Zn can lead to toxicity, which may manifest as reduced growth and yield, chlorosis, and reduced root and shoot biomass. The threshold range for Zn in plants is generally considered to be between 20-100 ppm (Alloway, 2012). Fe is also an essential micronutrient for plants and is involved in a wide range of physiological processes, including photosynthesis, respiration, and nitrogen fixation. The threshold range for Fe in plants is generally considered to be between 50-500 ppm (Barker and Pilbeam, 2015). Mn is an essential micronutrient required for photosynthesis, enzyme activity, and nitrogen metabolism. The recommended range for Mn in plant tissues is between 15-100 ppm (Nagajyoti et al., 2010). Cu is required in small amounts for plant growth and development, but excessive uptake can lead to toxicity. Symptoms of Cu toxicity in plants include reduced growth, leaf curling, chlorosis, and necrosis. The threshold range for Cu in plants is generally considered to be between 4-15 ppm (Awashthi, 1999; Nagajyoti et al., 2010). Ni is an essential micronutrient required for urease enzyme activity and seed germination. The recommended range for Ni in plant tissues is between 0.1-1 ppm (Allaway, 1968; Awashthi, 1999; Nagajyoti et al., 2010).

These essential heavy metals are required in some biological roles as they act as co-factor of enzymes, part of enzymes, while others are required for redox reactions in plants. Whereas some heavy metals have no known biological functions in plants; therefore, they are not required by the plants, known as non-essential heavy metals, Mercury (Hg), Cadmium (Cd) and Chromium (Cr) are some non-essential heavy metal examples (Wuana and Okieimen, 2011). The threshold levels for non- essential in plant tissue are typically lower compared to the essential heavy metals. The recommended maximum limit for mercury in edible plant tissue is 0.01 mg/kg for cadmium is 0.3-1.0 mg/kg and for chromium is 0.1-1.0 mg/kg, depending on the plant species (Organization, W. H, 2009). Heavy metal concentration limits in cereal crops according to the World Health Organization (FAO/WHO, 2017), European Union (EU) (Regulation, 2006), and United States Environmental Protection Agency (EPA, U. S, 2019) for various heavy metals can be seen in **Table 1**.

**Table 1:** Recommended concentration limit of heavy metals in different crops and plant species.

Heavy metal	Crop/plant species	WHO maximum limit (mg/kg)	EU maximum limit (mg/kg)	EPA maximum limit (mg/kg)
**Arsenic (As)**	Rice	0.2	0.2	0.01
	Leafy vegetables (spinach, lettuce, etc.)	0.1	0.1	0.4
	Root vegetables (carrots, potatoes, etc.)	0.1	0.1	0.4
**Cadmium (Cd)**	Rice	0.2	0.05	0.4
	Leafy vegetables (spinach, lettuce, etc.)	0.1	0.1	0.3
	Root vegetables (carrots, potatoes, etc.)	0.1	0.1	0.3
**Chromium (Cr)**	Leafy vegetables (spinach, lettuce, etc.)	N/A	0.5	1.1
**Copper (Cu)**	All crops	N/A	50	15-250
**Mercury (Hg)**	Rice	0.02	0.02	0.1
	Leafy vegetables (spinach, lettuce, etc.)	0.02	0.02	0.1
	Root vegetables (carrots, potatoes, etc.)	0.02	0.02	0.1
**Lead (Pb)**	All crops	0.3	0.1	0.1
**Zinc (Zn)**	All crops	N/A	100	N/A
**Iron (Fe)**	Wheat	–	–	500
	Rice	–	–	500
	Barley	–	–	500
**Manganese (Mn)**	Wheat	100	–	–
	Rice	50	–	–
	Barley	50	–	–

Provided by: WHO; FAO, Food and Agriculture Organization; EC, European Union Standards.

Both natural and anthropogenic activities expose heavy metals in the environment, but anthropogenic activities are the core cause of most of the heavy metal pollution in the atmosphere (Smiljanic et al., 2019). Weathering different types of rocks and minerals found in the Earth’s crust results in various heavy metals but usually within acceptable limits (Chatterjee and Chatterjee, 2000). There are two types of soil in which heavy metal pollution is produced, including point and non-point sources. Point sources are pollution from discrete source, such as pipes or effluent outfalls. Non-point sources are sources with no discrete source, and the pollutants enter the environment *via* many pathways (Rehman et al., 2008). Both natural and anthropogenic sources may be point or non-point in nature. Natural elements from a parent substrate reach the soils during pedogenetic processes. Heavy metals in soil depend on the parent substrate’s geology (Öncel et al., 2000). Fuel consumption with transportation, homes, and industries usually releases Zinc (Zn), Lead (Pb), Cadmium (Cd), and Chromium (Cr). Relatively high levels of Cadmium (Cd), Arsenic (As), Lead (Pb), and Nickel (Ni) are observed in the exhaust and non-exhaust releases from vehicles (Lee et al., 2021). The burning of coal releases Arsenic (As), Cadmium (Cd), and Lead (Pb) (Ali et al., 2019). A number of industries release their effluents that contain heavy metals directly into various water bodies from where the heavy metals enter soil and food chains (Rehman et al., 2008). Zinc (Zn), Iron (Fe), Lead (Pb), and Manganese (Mn) are released due to the burning of hair and effluents of the tanning industries (Hashem et al., 2017). Heavy metals can accumulate in plants when they are grown in contaminated soils or exposed to polluted air and water. When humans consume these contaminated plants, they can be exposed to high levels of heavy metals, which can lead to serious health problems. Heavy metals such as lead, cadmium, arsenic, and mercury are particularly concerning due to their toxicity and ability to accumulate in the body. When humans consume plants containing these heavy metals, they can experience a range of adverse health effects. For example, lead can cause neurological damage and developmental delays in children, while cadmium can damage the kidneys and increase the risk of cancer. Arsenic exposure has been linked to skin, lung, and bladder cancer, and mercury can cause neurological damage and developmental delays in children (Mudgal et al., 2010; Gall et al., 2015).

Plants possess a significant ability to absorb and store pollutants in their tissues. The mechanisms of metal uptake and transfer to different parts of plants have been the subject of various researchers (Ma et al., 2010; Rastogi et al., 2017; Ul Haq et al., 2020; Ihtisham et al., 2021). Heavy metals are usually present in the state of ions or precipitates in the soil that plants facilitate to induce the pH change of the soil and the production of chelators (Adamczyk-Szabela et al., 2015; Yang et al., 2022b). Essential and non-essential heavy metals have similar structural characteristics, which makes it difficult for plants to distinguish between the two metal classes. Thus, the root hairs ingest essential and non-essential metals from soil sap, where their concentration is usually higher than the epidermal cell sap. The soil sap enters the epidermal cells using a symplast pathway, which crosses into cortical cells *via* the plasmodesmata. The sap enters from cortical cells through the apoplast pathway to the stele through the plasmodesmata. The cell sap can move through the plasmodesmata and enter the root’s xylem cells (Meers et al., 2009; Tangahu et al., 2011). The plasma membrane of epidermal cells has several channel proteins and pumps for the uptake. These include a) proton pumps, which are special ATPases that use energy to generate electrochemical gradients, b) co and anti-transporters that uptake metals using electrochemical gradients that are generated by proton pumps, and c) carrier proteins that transfer ions into the cells (Ardestani and Van Gestel, 2013).

Heavy metals move upwards from the roots to the other sections of the plant along the xylem stream. Atmospheric heavy metals are usually released as aerosols, and vapors are filtered by the leaves from the atmosphere (Shahid et al., 2017). There are morphological traits, such as cuticle thickness, the stomatal, and the surface area of leaf (Barber et al., 2004; Larue et al., 2014) and physicochemical traits of the heavy metals, such as the density, size of the ion, and solubility of the metal ion (Xiong et al., 2014). A report shows that vegetables that grow near heavy industries have a higher content of heavy metals in their leaves (Shahid et al., 2013). The uptake of heavy metals occurs through the stomatal pores, which are cracks in the cuticle, ectodesmata, which are special channels in between the auxiliary cells and guard cells of the epidermis, and aqueous pores (Fernández and Brown, 2013). Many studies show that the foliar absorption of heavy metals is dose dependent, and there is a linear relationship between many heavy metals concentration in the air and their concentration inside the leaves (Kozlov et al., 2000; Bondada et al., 2004; Fernández and Eichert, 2009).

## 2 Impact of heavy metal on plant’s structural and functional biology

Plants undergo different morpho-physiological and anatomical changes during oxidative stress (Li et al., 2021; Li et al., 2022a; Noor et al., 2022; Sun et al., 2022). Heavy metals interact with plants in two ways. First, heavy metals compete with essential nutrients during root uptake from the soil, preventing plants from growing normally. Second, heavy metals enter the plant, disrupt its metabolism, and have toxic effects on its internal and external structure (Liza et al., 2020). Heavy metal concentrations above the permissible level will negatively affect plants directly and indirectly (Blaylock, 2000). The direct negative effects include inhibiting enzymatic activities *via* binding to the sulfhydryl group or a deficiency of certain metals in metalloproteins or metal protein complexes (Van and Clijsters, 1990). Another direct effect is the damage to cellular structures, such as chloroplast and mitochondria, due to oxidative stress (Jadia and Fulekar, 2009). High doses of certain heavy metals slow down the process of photosynthesis, transpiration, and the growth rate in various plants (Yadav, 2010). We will discuss the impact of heavy metal stress on plants’ structure and functional biology in detail in this review.

### 2.1 Morphological changes in plants

Heavy metals in plants can visualize themselves *via* visible damage to the epidermal tissues of the roots, stems, and leaves. A study noted a reduction in the leaf thickness because of the increased size of the bulliform and endodermis cells, which forced a decline in the size of the parenchyma cells (Alfaraas et al., 2016). Another study recorded a reduction in the leaf lamina size, root, and shoot length of *Shorea robusta* due to Cd, As, and Pb contamination (Pant and Tripathi, 2014). A reverse effect was observed with Cd and Pb in some plants. The plant length of different parts increased, but the volume of these organs decreased (Zhang et al., 2020; Wu et al., 2021). Heavy metals are toxic to plants, which causes chlorosis, slows down plant growth, and a reduces yield (Singh and Kalamdhad, 2011). A previous study, which assessed the micromorphological changes in Taraxacum officinale due to heavy metal toxicity, observed a reduction in the leaf thickness and many more spaces among the cortex and other cells compared to the control group (Bini et al., 2012). An increase in the diameter of the root and shoot, the enlarged trichomes and salt glands, and a variation in the number of stomata are some of the morphological changes observed in *Catharanthus roses* grown in heavy metals contaminated soil (Soumya et al., 2022). Zn and Cd phytotoxicity in *Brassica juncea and Phaseolus vulgaris* causes a reduction in growth and development (Prasad et al., 1999). Morphological characteristics, fresh and dry weights, shoot/root length, leaf area, and leaf count of *Parthenium hysterophorus* were reduced due to the negative impact of Pb and Cd (Ejaz et al., 2022). Higher levels of Cr negatively impact the plant’s total biomass, root and shoot growth (Alsafran et al., 2022). Dandelion (*Taraxacum officinale*) growing in contaminated soil, showed reduced leaf thickness and poor structural pattern of leaves and roots (Maleci et al., 2014).

### 2.2 Anatomical modifications in plants

The researchers studied dissimilarities in the internal structures of the plant’s roots, stems, and leaves in response to heavy metals (Gomes et al., 2012; Noreen et al., 2019; El-Shabasy, 2021). A detailed overview of heavy metal’s effects on the anatomy of various organs of plants is provided below.

#### 2.2.1 Root and stem anatomy

Heavy metals penetrate the plants from the soil *via* the roots. The roots, therefore, receive more harmful effects than other parts of the plant. Parenchyma collapsed in a paddy plant’s roots due to Pb and Cd-Pb treated plants (Alfaraas et al., 2016). Cd caused damage to the root’s endodermis, epidermis, and cortex on the tissue level in non-resistive genotypes of rice plants, whereas no visible damage was observed in the resistant plants (Li et al., 2014). There was a 20-30% reduction in the root parenchyma and the size of parenchyma cells with a combined treatment of Cd-Cu ((Kasim, 2005). The visible negative consequences of Cd toxicity were noted on the lateral root, stem primordia and, general root architecture (Ronzan et al., 2018).

There are visible changes in the structure of various cell organelles and the pattern of cell division in reaction to heavy metals on the cellular levels. The researchers obtained the results for reducing cell division in apical root meristems of *Lamina minor* treated with Pb (Samardakiewicz and Woźny, 2005). Also, the chromosome morphology, such as anaphase bridges and chromosome stickiness, were affected similarly, which caused a reduced cell division in apical meristems of the roots in *Helianthus annuus* due to Cd toxicity (Jiang et al., 2000). A reduction in cell division due to Ni and Pb toxicity was observed for various other plants. The restricted cell division in the apical and lateral meristem of the roots is the interaction of these metals with chromosomes during the cell division (Kozhevnikova et al., 2007). They also observed the structural modification in root hairs, cell walls, and the vacuoles of the cells in the roots of cotton plants. Cell vacuoles generally increase in size, probably for the accretion of heavy metals that are absorbed by the roots (Daud et al., 2009). The researchers also observed structural modifications in the cell walls, vacuoles, and root hairs of the cell in the roots and stems of cotton plants. The thickening of the cell wall in *Vicia faba* was recorded in the roots and stem cells in heavy metal stress plants (Ronzan et al., 2018). The thickening of the cell wall is a resistant mechanism against heavy metal stress. The stems of some plants show xerophytic adaptations due to heavy metal toxicity, such as a thick cuticle over their epidermis, a thick cortex with a stone like an appearance, and general structural modification in the vascular bundles (Raju and Ramakrishna, 2021).

#### 2.2.2 The anatomy of leaves

A leaf is the most fragile organ of the plant, which is severely damaged by environmental pollution (Dickison, 2000). Heavy metals can enter the leaf *via* the stomata or translocate from the roots *via* the stem. Heavy metals in the interior of the leaves have serious consequences for the leaf tissue as well as at the cellular level (Dickison, 2000; Yabanli et al., 2014). Due to the Cd treatment, the chloroplast was the most affected organ in Salix purpurea and Phragmites australis at the cellular level (Hakmaoui et al., 2007). Several grana and thylakoid membranes were negatively affected due to the leaf’s heavy metal build-up. The cell showed a less developed vacuolar system at a high absorption of Cd (Malik et al., 1992; Ciscato et al., 1997; Hakmaoui et al., 2007). Heavy metal exposure in high concentrations also negatively affects cell division and the differentiation of newly developing leaves in addition to the chloroplast and vacuolar system (Cheng, 2003; Li et al., 2014). The leaf showed a thick cuticle with a wax deposition and expanded mesophylls (Raju and Ramakrishna, 2021).

### 2.3 Physiological changes in plants

Heavy metal stress has a great impact on the physiology of the plant. The researchers discovered that heavy metal accumulation in plants reduced biomass, chlorophyll, and photosynthesis activity, whereas proline and antioxidant enzymes increased. Various studies showed that the plant’s soluble sugar content decreases as the concentration of heavy metal stress increase, particularly in crops. (Hemalatha et al., 1997) (Rascio and Navari-Izzo, 2011). Heavy metals influence many biological activities, including denaturing several enzymes (Ghori et al., 2019). The hyper activity of many enzymes, which include glucose-6-phosphate dehydrogenase and peroxidases, are linked to heavy metal toxicity in plant leaves (Van and Clijsters, 1987), which ultimately affects the stability of the cell membrane. The heavy metal accumulation of Ni, Cd, Cr, Ar, Pb, Ni, and others disturbs the plants’ metabolic processes and physiological functions (Singh and Aggarwal, 2011). The proline amount in plant species increases under heavy metal stress, but the chlorophyll concentration decreases (Ahmad et al., 2021). Excess zinc inhibits the germination of cluster beans (*Cyamopsis tetragonoloba*) and its growth, sugar, amino acid, chlorophyll, and carotenoid content (Manivasagaperumal et al., 2011). Zn causes phytotoxicity in plants if it exceeds the required nutrient level (Vries et al., 2007). A high Zn level in soil restrains numerous plant metabolic activities, which results in fast growth (Choi et al., 1996). It has been demonstrated that cadmium toxicity in plants decreases the ATPase activity in the cell membranes of wheat and sunflowers (Fodor et al., 1995; Tang et al., 2020). Under Cd stress, plants experience a decrease in physiological activities such as stomatal opening, leaf moisture content, and transpiration, which causes osmotically stressed conditions due to which plants experience severe physiological disorders (Alsafran et al., 2022; Javad et al., 2022). Cd also causes symptoms, such as chlorosis, oxidative stress, and the darkening of the roots, which can all be fatal (Di Toppi and Gabbrielli, 1999), (Mohanpuria et al., 2007), (Mohanpuria et al., 2007). Cadmium above the threshold level may cause quick death and disturb the enzymes structure and function in plants and microorganisms (Prasad et al., 1999; Yang et al., 2022c). A change in the efficiency of the catalytic enzymes in *Phaseolus vulgaris* occurs due to a high concentration of Cd and Zn (Van et al., 1988) (Romero‐Puertas et al., 2004). Lead is a harmful metal that causes necrosis, chlorosis, limited plant growth, and a low yield (Malar et al., 2014). A study on the rate of cell division in heavy metals shows that when the concentration of the heavy metals increased, the cell division exponent decreased, demonstrating a negative effect of the heavy metal on cell division (Duan and Wang, 1995). Nickel is very toxic at a high level and is currently being studied due to its significant deposition in sediments across the globe (Singh et al., 2017). Numerous physiological changes, which include chlorosis and necrosis, are caused by too much Ni in the soil in many plant species, which is most notable in rice (Zornoza et al., 1999) (Rahman et al., 2005) (Das et al., 1997). Ni at toxic levels adversely affects plants by interfering with a variety of physiological processes, including nutrient deficiency, growth parameters, enzyme activities, and photosynthesis. (Naz et al., 2022) Mercury at high concentrations is highly toxic to plant cells. Different studies on mercury (Hg) toxicity demonstrated that it could cause visible damage and physiological issues in plants. It attaches to water transfer proteins, which results in the stomata shutting and blocking the water passage in plants. (Zhang and Tyerman, 1999) (Zhou et al., 2007). According to a study, high mercury concentrations slow mitochondrial activity by triggering reactive oxygen species (Messer et al., 2005) (Sheetal et al., 2016). It was observed that an excess amount of cobalt decreased the chlorophyll content in plants by studying high concentrations of cobalt in cauliflower leaves. Manganese phyto-toxicity causes necrotic and chlorosis on the leaves and stems. Another warning sign is a crinkled leaf, which develops on young leaves, stems, and petiole tissue. (Wu, 1994) (Wu, 1994) (Elamin and Wilcox, 1986). A high concentration of Mn in plants shortens the shoot and root length (Arya and Roy, 2011). Mn toxicity was examined in peas (*Pisum sativum*), and it was discovered that chlorophyll a and b concentrations, the relative growth rate, and the photosynthesis rate decreased with an increased Mn level (Doncheva et al., 2005). However, slower plant development and a decrease in the chlorophyll content were observed in tomatoes (*Lycopersicon esculentum*), which was recorded by (Shenker et al., 2004). Arsenic inhibits growth, and it results in the discoloration and the wilting of plants (Cox et al., 1996). Arsenic similarly inhibits the plant height and leaf area in *Oryza sativa* (Marin et al., 1993; Abedin et al., 2002; Pan et al., 2023). Plants that grow in polluted land produce more proline. which is a survival mechanism towards heavy metal stress, but the amount of carotenoids and chlorophyll decreased (Ahmad et al., 2023). Heavy metal inhibits plant growth by altering its physiological and biochemical processes. It is consequently obvious from several research findings that heavy metal contamination has a severe impact on the physiology of plants.

## 3 The role of plant genomics and transcriptomics under heavy metal stress

### 3.1 Genomics

Specific structural genes control plant tolerance, so it is necessary to recognize, validate, and characterize the genes linked to heavy metal stress. Plant stress genes are generally divided into two groups: early functional and delayed functional. The early functional genes become active rapidly but only briefly, whereas the delayed functional genes are slowly and consistently induced. The ATPase (HMA) gene family is linked to the accumulation of heavy metals, transportation, and effective resistance in plants. HMAs can be divided into two main subgroups: the Pb/Zn/Cd/Co P1B-ATPase and the Ag/Cu P1B-ATPase, based on their preference for specific metal substrates (Axelsen and Palmgren, 2001). Eight P_1B_-ATPases were discovered in *A. thaliana* (Williams and Mills, 2005). P_1B_-type ATPases was additionally discovered in *Triticum aestivum, Hordeum vulgare, Arabidopsis halleri, and Thlaspi caerulescens* (Deng et al., 2013). Different HMA gene expressions in different tissues protect *Populus trichocarpa* from the heavy metal stress of Ag, N, Cd, Cu, Zn, Pb, Mn, and Co (Li et al., 2015). HMA8 genes have been expressed in high levels in heavy metal stress conditions, in HMA1 and HMA4 leaves, and in the HMA5.1 roots. Heavy metal uptake and more expression of the genes have a direct correlation. For instance, HMA3 overexpression causes improved Cd accretion in plant parts (Morel et al., 2009). Over expression of HMA5 has similarly been found in *Oryza sativa* growing under high Cu contaminated soil (Deng et al., 2013). *Aeluropus littoralis* regulates the H^+^-ATPase gene to control its potential for the remediation of Pb and Hg metals. (Jam et al., 2014). High concentrations of Cd resulted in overexpression of the gene family serine acetyltransferase (SAT) in *Arabidopsis thaliana* (Howarth et al., 2003).

### 3.2 Transcriptomics

Numerous studies have been conducted on transcriptomics to understand gene expression in heavy metals. Transcription factors (TFs) come from multigenic groups and control the expression of numerous genes, known as the main regulators (Hu et al., 2022). TFs attach to the distinct locations of cis-acting elements in gene promoters to control gene expression (Wray et al., 2003). Many TFs groups have been identified that control how plants react to heavy metal stress, which includes E2F-DP, E2F-DP, AREB/ABF, CCAATDR1, MYB, CCAAT-HAP3, DREB1/CBF, EMF1, MADS, AP2/EREBP, C2C2-Dof, CCAAT-HAP5, bHLH, C2H2, C3H, C2C2-YABBY, C2C2-Gata, ABI3VP1, ARF, C2C2-CO-like, ARID, CPP, CCAAT- HAP2, SBP, WRKY, bZIP, HSF, MYC, HB, AtSR, TUB, and NAC (Singh et al., 2002; Shameer et al., 2009; Noman et al., 2017). The basic leucine zipper (bZIP) in *Arabidopsis thaliana* and *Brassica juncea* transcription factors are activated in response to Cd stress (Ramos et al., 2007). TFs were additionally discovered in *Arabidopsis halleri* under Cd stress (Weber et al., 2006). *A. thaliana* exposed to Cd toxicity had two additional TFs, ERF1 and ERF5, induced by the AP2/ERF superfamily (Herbette et al., 2006). *Brassica napus* under Cd stress induced different transcripts, such as miR156, miR171, and miR396a (Zhou et al., 2008). Various levels of Cd stress may carry on the differential TF expression in these plants. miR166 was discovered to be downregulated in modified miRNA due to Cd stress, whereas miR171, miR529, miR319, and miR393 were observed to be highly expressed in *Medicago truncatula* (Zhou et al., 2008). miR529, miR319, MiR171, and miR393 were discovered to be upregulated under high Hg stress in *Medicago truncatula* (Zhou et al., 2008). Other researchers reported that miR398 is downregulated in plants under Hg and Cd (Kuo and Chiou, 2011; Min Yang and Chen, 2013). 18 different miRNAs were discovered in *Oryza sativa* under As stress due to differential expressions (Liu and Zhang, 2012). A scientist reported 69 miRNAs in *Brassica juncea* (Srivastava et al., 2012). The plant’s growth was positively influenced by altering the expression of miR167, miR319, and miR854 *via* the artificial application of JA and IAA in another study (Gupta et al., 2014). Rice similarly showed a differential expression of seven miRNAs, which are encrypted genes for the transportation of nutrients, transcription factors, induce apoptosis, phytohormones equilibrium, and cell expansion under oxidative stress (Li et al., 2010).

## 4 Molecular level response mechanisms of plants

Heavy metal toxicity in plants results in the induction of cellular defense strategies, such as transportation and detoxification of heavy metals in the vacuole, which produces heavy metal transporters, amino and other organic acids, antioxidants, and phytochelatins (Noor et al., 2022). Plants need many metal ions for various biomolecules in plants. Some heavy metals, which are required in small quantities, are required by enzymes as co-factors and other biomolecules. However, non-essential heavy metals negatively affect plants by restricting vital functional groups or displacing important metal ions in the biomolecules (Parmar et al., 2013).

### 4.1 Heavy metal transporters

**Figure 1:**
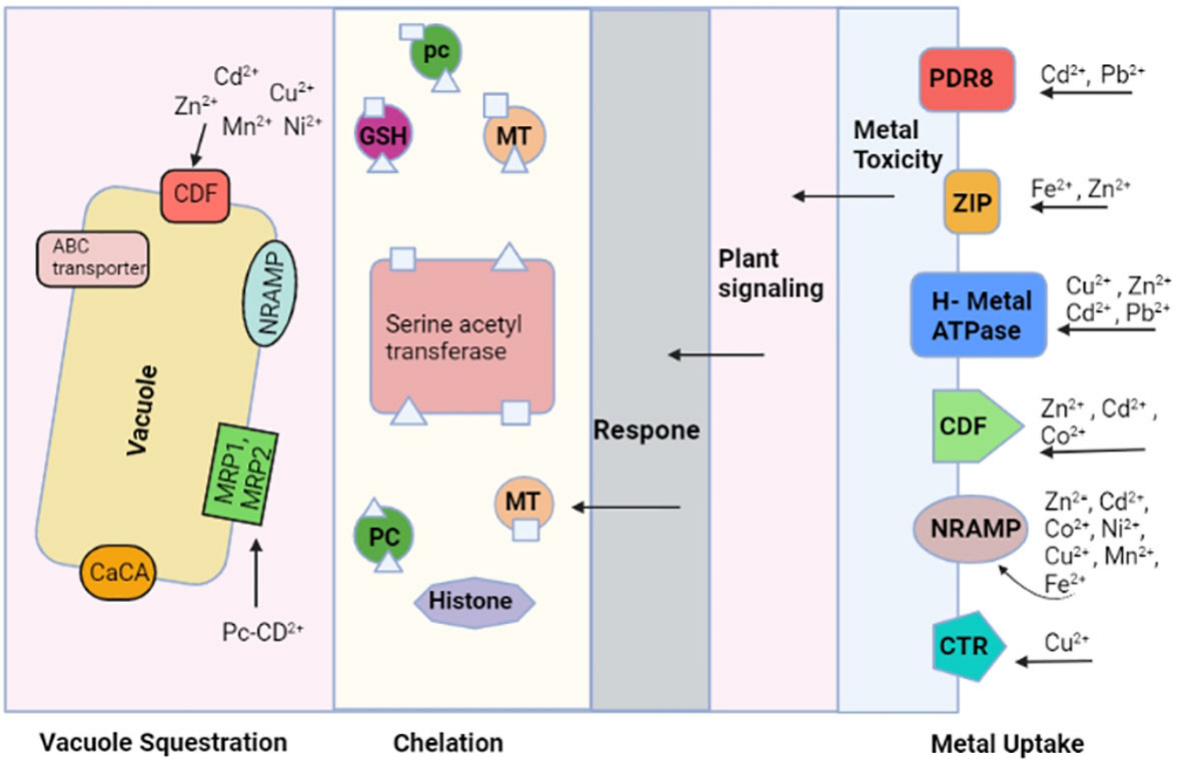
The biomolecules that are involved in uptake of heavy metals, chelation, and heavy metal sequestration/compartmentalization are shown. Many metal ion transporters are involved in this process. A high concentration of heavy metals in the cell initiates a defense response whereby heavy metal chelators are released, attached with heavy metals, and transferred in the vacuole.

Heavy metal transporters are believed to play an important role in plants, which implies that these types of transporters might be vital in the resistance to heavy metals, induced toxicity. These metal ion transporters include CPx-type ATPases, zinc-iron permeases (ZIP), and macrophage protein (Nramp) (Williams et al., 2000). These transporters are believed to be involved in obtaining heavy metals for vital cellular functions and regulating them (Parmar et al., 2013). Another family of proteins is Nramp-, which is involved in the uptake of Fe and Cu-, and it has been discovered to increase the Cd sensitivity if the related gene is overexpressed in certain heavy metal stress conditions (Thomine et al., 2000). Many ZIP family members have been identified so far, and at least 15 genes of the ZIP family members are in the genome of *Arabidopsis thaliana.* The ZIP family transporters help in Zn, Cd, and Co transportation. A study on zinc transporters in *Arabidopsis thaliana* suggests that protein assists the Zn sequestration (Van Der Zaal et al., 1999). Many intracellular transporters, which include HMA, ABC, CDF, NRAMP, and CaCA, participate in the compartmentalization of heavy metals. Chelated metals inside the vacuole depend on the activity of the two families of ABC transporters, known as multidrug resistance associated proteins (MRP) and pleiotropic drug resistance proteins (PDR). In addition, PC-Cd (phytochelatin-cadmium) complexes are transported by HMT1 transporters, found in the tonoplast, and tonoplasts contain CaCA and NRAMP transporters, which help in the shift of heavy metals from the cytosol to the vacuole. Many eukaryotes have CDF transporters. They have been observed to transport Cd, Ni, Fe, Mn, Co, and Zn metal cations from the cytoplasm to the vacuole, illustrated in **Figure 1** (Krämer et al., 2007; Montanini et al., 2007; Peiter et al., 2007).

### 4.2 Amino and other organic acids

Certain other essential biomolecules have been discovered to help with the resistance against heavy metal toxicity in addition to heavy metal transporters. Some of these biomolecules comprise of amino acids, organic acids, phytochelatins, and metallothioneins (Grill et al., 1987; Kägi, 1991; Homer et al., 1997; Rauser, 1999). Several amino acids in combination with other organic acids in plants help in the chelation of heavy metals. The amino acids include Ile and Trp (Pb), Leu and Gly (Cd), succinic, oxalic, butyric, and citric acid (Hg), Ser (Hg), Glu and Trp (Ni), Gly, Leu, and Asn (Se), Ser, Leu, and Asp (Cr), organic acids, malic acid and malonic acid (Cd), citric and malonic acid (Se), malonic acid (Pb), Thr and Asp (Sn), malic and oxalic acid (Cr), malic and malonic acid (Ni), maleic, and malonic and malic acid (Sn) (Kocaman, 2022). The accumulation of asparagine against Zn toxicity was observed in the roots of *Deschampsia cespitosa*. The possible mechanism involves the Zn-Asparagine complex formation to detoxify Zn (Smirnoff and Stewart, 1987). A number of Brassicaceae members have shown high concentrations of free histidine in the xylem response to an Ni accumulation (Krämer et al., 1996; Kerkeb and Kramer, 2003). The proline concentration rose in response to the Ni toxicity in three plants, which included *Walsura monophylla, Phyllanthus palwanensis*, and *Dechampetalum geloniodes* (Homer et al., 1997). A major portion of Zn was observed to be bound by asparagine and proline in tomato and soya bean xylem sap (White et al., 1981). The plant releases particular amino acids in response to heavy metals, as shown in **Table 2**.

**Table 2:** shows a number of amino acids that are released by plants against a particular heavy metal.

Plants	Heavy metals	Amino acids							
		Proline	Histidine	Asparagine	Aspartate	Threonine	Cysteine	Lysine	Ref.
*Walsura monophylla*	Ni	✓	–	–	–	–	–	–	(Homer et al., 1997)
*Nepeta cataria*	Pb	✓	–	✓	–	–	–	✓	(Zhou et al., 2020)
*Solanum lycopersicum*	Cu	–	–	–	✓	✓	–	–	(White et al., 1981)
*Phyllanthus palwanensis*	Ni	✓	–	–	–	–	–	–	(Homer et al., 1997)
*Solanum lycopersicum*	Cu	–	–	–	✓	✓	–	–	(White et al., 1981)
*Alyssum lesbicum*	Ni	✓	–	–	–	–	–	–	(Krämer et al., 1996)
*Brassica juncea*	Ni	–	✓	–	–	–	–	–	(Parmar et al., 2013)
*Deschampsia cespitosa*	Zn	–	–	✓	–	–	–	–	(Smirnoff and Stewart, 1987)
*Phyllanthus palwanensis*	Ni	✓	–	–	–	–	–	–	(Homer et al., 1997)
*Solanum lycopersicum*	Zn	–	✓	–	–	–	–	–	(White et al., 1981)
*Arabidopsis thaliana*	Cd	–	–	–	–	–	✓	–	(Domínguez‐Solís et al., 2004)

### 4.3 Phytochelatins

Plants and fungi release unique metal binding peptides called phytochelatins (PC) under heavy metal stress. PCs are an oligomeric form of glutathione with the attribute reappearance of the (-Glu-Cys)n-Gly [(PC)n], where n = 2–11 dipeptides of glutamate and cysteine (Ahmad et al., 2019a). Several heavy metals form PC complexes, but the most abundant ones are the PC complexes that involve Cd^+2^ and Cu^+2^. Other relatively less abundant PC complexes involve Pb, Hg, and Zn (Thumann et al., 1991; Mehra et al., 1995; Maitani et al., 1996; Mehra et al., 1996). PCs are produced against heavy metals stress of Ag, Hg, Cu, Pb, Zn, and As. Heavy metals stress boost the PCs genes (LsPCS1) appearance in some plant species (He et al., 2005). The PCS1 gene in wheat was responsible for tolerance against Cd toxicity, and this gene could be used in the preparation of transgenic crops for heavy metal phytoremediation (Khan et al., 2020).

## 5 Heavy metal induced cell signaling in plants

Plants interact with heavy metals in two ways. First, plants are harmed by heavy metals. Second, they develop resistance mechanisms against them (Asati et al., 2016). Plants have many defense mechanisms against heavy metals. The sensing of heavy metal stress by plants initiates a number of responses on molecular and biochemical levels (Jalmi et al., 2018). Plants have three signaling pathways: the MAPK cell signaling pathway, calcium signaling, and hormone signaling in heavy metals (Jalmi et al., 2018).

### 5.1 MAPK pathway in heavy metal stress

A conserved evolutionary cell signal transduction module, called mitogen-activated protein kinase (MAPK), is involved in directing the extracellular cell signals to the nucleus to start suitable cellular responses. There are three components in the MAPK cascade, which include a) MAPK kinase (MAPKKK), b) MAPK kinase (MAPKK), and c) an MAPK. These components are connected through phosphorylation (Sinha et al., 2011). The MAPK signaling pathway is involved in mitosis especially during phragmoplast synthesis (Calderini et al., 1998; Yang et al., 2022a). The MAPK signaling transduction is extremely important to basic physiological functions, such as cell cycle regulation, abiotic stress signaling, and the defense mechanism (Tena et al., 2001). The accurate mechanism behind activation of this specific signaling pathway required a lot of investigation, but the heavy metal ligands and the reactive oxygen species (ROS) are the main factors that are responsible among the abiotic factors (Jonak et al., 2004; Smeets et al., 2013; Jalmi and Sinha, 2015). Heavy metals, such as Cd, Cu, and As induce MAPK signaling activation (Jonak et al., 2004; Yeh et al., 2007; Ding et al., 2011; Rao et al., 2011; Smeets et al., 2013). However, very limited literature is still available about the response produced by other elements, such as Fe, Pb, and Zn. The exact mechanism against particular heavy metals is not yet understood in addition to this, but the researchers have investigated the pathways that are involved in a number of species. The MAPK cell signaling pathway is especially important in regards to mitigating heavy metal stress in number of plants. The exposure of *Medicago sativa* seedling, Cd, and Cu stress trigger four distinct MAPKs, which include a) SIMK, b) MMK2, c) MMK3, and d) SAMK. All four MAPKs increased their activities with an increase in the concentration of CdCl_2_ and CuCl_2_ (Jonak et al., 2004). MPK6 and MPK3 are the best known MAPKs in Arabidopsis that trigger stimuli, such as CdCl_2_ and CuSO_4_ (Asai et al, 2002; Pitzschke et al., 2009; Liu et al., 2010; Ahn et al., 2011; Beck et al., 2012). *Oryza sativa* increases the transcription of OsWJUMK1 (OsMPK20-4 homolog), OsMSRMK3 (OsMPK7 homolog), and OsMSRMK2 (OsMPK3 homolog) when treated with Cd and Cu (Rao et al., 2011; Beck et al., 2012). Other heavy metals also similarly induce MAPK cascades in Cu and Cd, but their mechanism is still not very widely investigated. For example, an Al ion sensitive yeast mutant showed an over expression of the MAPK gene, which suggests an alliance of the MAPK gene with an Al confrontation (Schott and Gardner, 1997). Al resistance is achieved in wheat roots with the induction of a 48kDa MAPK signaling transduction. This shows a link between Al stress and MAPK activation (Mossor-Pietraszewska, 2001). Myelin basic protein (MBP) was found to be activated in rice by a 42kDa MAPK, which is due to iron stress. Pre-treatment with glutathione (GHS) of the root apical cells in rice decreased the apical cell’s death and reduced ROS-induced MAPK signaling (Tsai and Huang, 2006). The SIMPAK3 gene was significantly induced in tomatoes under Cd^2+^ stress. This strategy would increase the leaf’s chlorophyll content and the root’s biomass along with increased root activity, which all helped in Cd stress (Muhammad et al., 2019). Several biomolecules activate the MAPK pathway under heavy metal stress. These biomolecules include nitrogen oxide (NO), reactive oxygen species (ROR), and various plant hormones. such as auxins, ethylene, and abscisic acid (ABA) (Li et al., 2022b).

### 5.2 Calcium signaling under heavy metal stress

The Ca^+2^ signaling pathway is very complex in nature with various biomolecules that have varied roles in this pathway. Let’s first look into the components of this signaling pathway and then the significance of this signaling pathway in heavy metal stress. The sensor proteins, such as calcineurin B-like protein (CBL)-CBL, calmodulin-like proteins (CMLs), calcium dependent protein kinases (CDPKs), calmodulins (CaMs), Ca^2+^/CaM dependent protein kinases (CCaMKs), and interacting protein kinase (CIPK) modules identify the signatures of Ca. This results in physiological responses, such as metabolic pathways, ion transport, and gene regulation (Zeng et al., 2015; Kudla et al., 2018). The second phase consists of the responding molecules, such as the CIPKs and CDPKs. This type of signaling helps develop tolerance towards various stresses (Tripathi et al., 2009; Li et al., 2012; De La Torre et al., 2013). Several researchers reported ease in heavy metal stress in plants when Ca^2+^ was exogenously applied. Treatment with Cd has been shown to enhance the antioxidant enzyme activity, which includes the antioxidant enzyme activity of ascorbate peroxidise and glutathione reductase, which a reduction in the activities of these enzymes was achieved under exogenous application of Ca^2+^ (Ahmad et al., 2015). The application of Ca^2+^ to a sesame plant induced the upregulation of the acquired systemic tolerance system, such as antioxidant enzymes and lipid fractions to protect the membrane integrity (Makadia and Siegel, 2011; Abd_Allah et al., 2017; Chen and Wang, 2021). Some studies examined the effect of the exogenous Ca^2+^ application on the toxicity of heavy metals in plants, but the exact mechanism of the signal transduction through the calcium signaling pathway is still not very clear. Researchers have also identified potassium (K) as a regulator of calcium (Ca^2+^) signaling pathways (Assaha et al., 2017; Johnson et al., 2022).

### 5.3 Hormone signaling under heavy metal stress

Phytohormones are tiny molecules, usually derived from secondary metabolities, used in biological processes like cell division, cell differentiation, cell elongation, growth and metabolism (Jaillais and Chory, 2010; Davies, 2012; Zluhan-Martínez et al., 2021). Several plant hormones are being produced in various plant organs under different conditions in varied concentrations. The most prolific of these hormones are auxin (IAA), cytokinins, abscisic acid, gibberellin, ethylene and brassinosteroid. A brief discussion of two of these hormonal signaling pathways and their importance in heavy metal stress is examined in the following passages.

#### 5.3.1 Auxins

Auxin (Indole-3-acetic acid; IAA) is a vital hormone in plant growth and expansion. There are a number of important hormones within the auxin family, such as Indole-3-butyric acid (IBA), IAA, and NAA. Auxin helps plants in regards to creating a response to heavy metal toxicity by regulating its biosynthesis, degradation, signaling, and transport (Potters et al., 2007). Auxin plays significant functions in the root development both in normal and stress conditions. An important protein, PIN1, has been reported to influence the redistribution of auxin under Cu stress both in meristematic and elongation zones in the primary root of *Arabidopsis thialana* (Yuan et al., 2013). Several studies show that the endogenous synthesis of auxins is affected by heavy metals stress. These reports showed heavy metals stress correlations with auxin biosynthesis (Srivastava et al., 2013). It was observed that Cd induced nitrogen oxide (NO) concentration inhibits auxin transport under Cd stress, and it causes a reduction in the meristem size of the root. NO is also important in the auxin signaling pathway in Cd stress (Pető et al., 2011; Yuan and Huang, 2016). Heavy metal accumulation is generally a main aspect in reducing endogenous auxin production. *Brassica juncea* in heavy metal stress, which decreases the endogenous production of three auxins, was noted (Srivastava et al., 2013). The IAA production was similarly disturbed due to the Cd stress in barley roots (Zelinová et al., 2015).

The exogenous application of auxin improved tolerance in aux1 As toxicity in transformed plants implies the vital function of auxin transportation and signaling method in heavy metal stress (Krishnamurthy and Rathinasabapathi, 2013). An increase in the roots and stem growth of a sunflower was observed under moderate Pb stress after the addition of IAA (Liphadzi et al., 2006). A comparative study was conducted on the effect of L-TRP, which is an ancestor of auxin, on the seedlings of *oryza sativa* grown in Cd polluted soil. The study noted that better growth and yield was obtained in the L-TRP treated seedlings compared to the control group (Farooq et al., 2015). A number of other researchers noted similar synergistic effects of exogenously applied auxins and their precursors on heavy metals tolerance in plants and their possible use in regards to enhancing the phytoremediation capacity of plants, but the exact mechanism behind the better tolerance, which is due to the exogenous application of the hormone, is still not clearly understood. The possible mechanism may involve an association between the miRNAs and auxins during heavy metals stress (Srivastava et al., 2013). It is necessary to mention that a complex interaction is concerned with the endogenous synthesis of auxins under heavy metals stress with a possible crosstalk between various signaling pathways. Further exploration of the subject will help in regards to understanding the underlining mechanism of the auxin signaling pathway and its role in heavy metals tolerance in various plants.

#### 5.3.2 Abscisic acid

**Figure 2:**
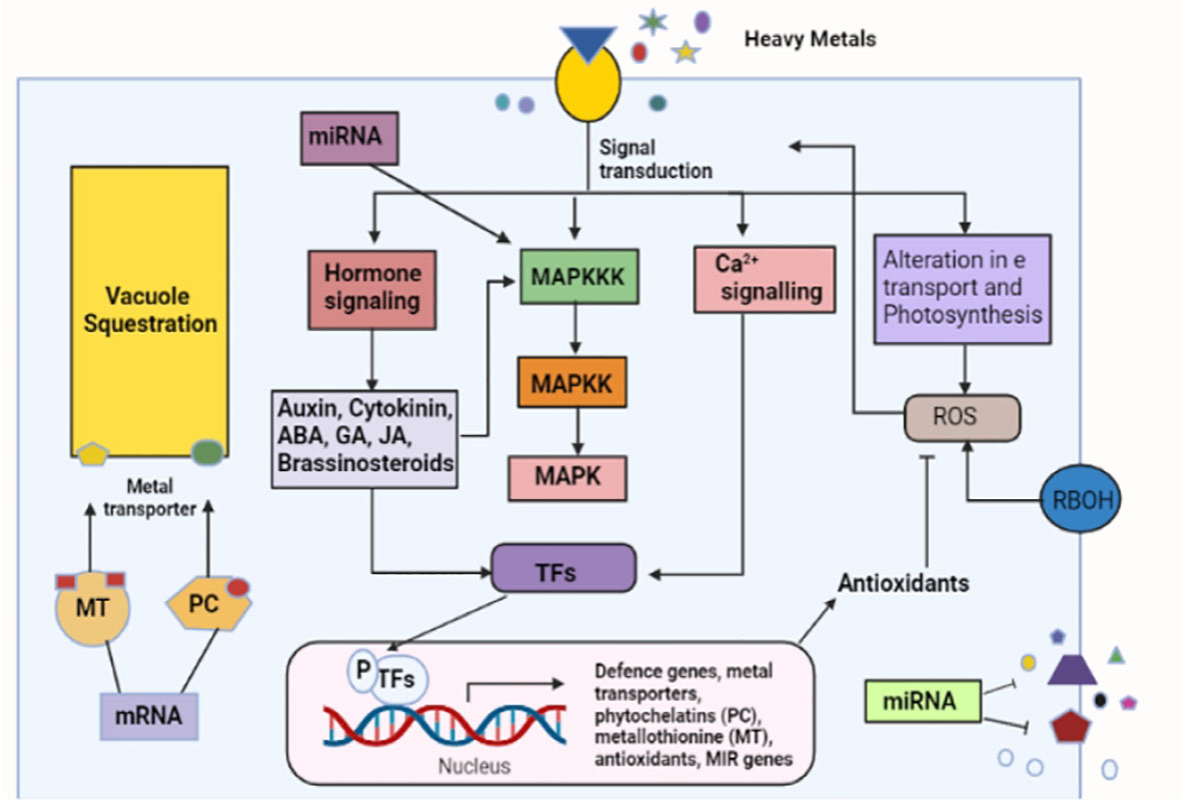
Crosstalk of the signaling pathways and the response created under heavy metals stress. The figure shows a number of signaling components working during heavy metal stress. Firstly, a high concentration of heavy metals is sensed, which initiates a cell signaling network that causes the activation of various metal responsive transcription factors.

The abscisic acid (ABA) hormone plays an important role in different stages of plants, such as seed dormancy and ripeness (Nambara et al., 2010). ABA also helps tolerate many environmental stresses, such as drought (Leung and Giraudat, 1998). The ABA signaling pathway controls abiotic stress (Bartels and Sunkar, 2005; Danquah et al., 2014). The concentration of abscisic acid increases with an increase in abiotic stress, which indicates that the plant cells can settle in the harsh environmental conditions as necessary. ABA signal transduction comprises a core signaling pathway that has Snf1-related protein kinases 2 (SnRK2s), type 2C protein phosphatases (PP2Cs), and PYL ABA receptors (Ng et al., 2014). The researchers show that ABA concentration increases in response to heavy metal toxicity (Rauser and Dumbroff, 1981; Poschenrieder et al., 1989). High amounts of ABA were observed in *Typha latifolia* and *Phragmites australis* due to heavy metal exposure (Fediuc et al., 2005). Similar results were obtained by (Stroiński et al., 2010) for potato tuber and by (Kim et al., 2014) for rice. A solution of Hg, Cd, and Cu was applied separately during the growth of wheat seeds, and the ABA level increased with a high accumulation of heavy metals (Munzuroğlu et al., 2008). The cucumber seedlings observed a reduction in growth and an increased level of ABA under Cu and Zn stress (Wang et al., 2014). Contamination show the expression of ABA synthesis related genes in *Oryza sativa*, such as OsNCED3 and OsNCED2, which is the directive of four ABA signaling genes. A entire genome study of rice root bare to vanadium (V) showed a strong demonstration of ABA signaling related genes (Lin et al., 2013). The transcriptional control of ABA signals transduction during cucumber seed germination under Cu and Zn stress showed that in total nine PLY, two SnRK2, and three PP2C genes were involved in the ABA signal transduction (Wang et al., 2014). The above mentioned pathways can be seen in **Figure 2**.

### 5.4 Mechanism of tolerance against heavy metals toxicity

We conclude that plants use the following mechanisms for tolerance against heavy metals in light of the above discussion.

#### 5.4.1 Role of plant’s roots in heavy metal uptake

Plants have evolved several mechanisms to create barriers and reduce the uptake of heavy metals through their roots. One such mechanism is exclusion, where plants restrict the entry of heavy metals into the root system through the formation of Casparian strips, which are suberin-like layers that surround the endodermal cells of the root. This prevents the passage of heavy metals into the vascular tissue. Plants can also release organic acids and other compounds from their roots that can react with heavy metals in the soil, forming insoluble complexes that are less available for uptake by the plant (Fahr et al., 2013). Some plants can actively pump heavy metals out of their roots using ATP-dependent transporters, a process called active efflux. Additionally, plants can reduce the uptake of heavy metals by competing with other nutrients for absorption sites on the root surface.

#### 5.4.2 Function of plant cell wall in metal tolerance

It has been reported that bivalent and trivalent metal cations bind to various functional groups, such as -OH, –SH, and –COOH to restrict the heavy metal to the cell wall, which is due to the presence of the carboxyl group in pectin of the cell walls (Mehes-Smith et al., 2013). Several heavy metals are known to accumulate in the cell wall of the epidermal cells of *humilis* and *Silene vulgaris ssp*, and the metals usually bind to pectin or silicates (Bringezu et al., 1999). The cell wall acts as a physical blockade to the entrance of heavy metals in the cell. However, it is interesting to note that it is still not clear how heavy metals are restricted in the cell wall.

#### 5.4.3 Plasma membrane as a barrier towards heavy metals entrance to the protoplast

The plasma membrane contains a number of heavy metal transporters, which are very helpful with the tolerance against heavy metal toxicity. They both act as channels for the intake of essential and non-essential heavy metals as well as induce sensitivity against heavy metals toxicity (Solioz and Vulpe, 1996; Williams et al., 2000; Parmar et al., 2013). The metal transporters are of practical importance for phytoremediation, and they are important for the tolerance against heavy metals toxicity (Ahmad et al., 2019b).

#### 5.4.4 Phytochelation

The excretion of phytochelatins by plants against heavy metals is the best strategy that plants use. High affinity ligands, such as PCs, bind to metal cations to immobilize them and restrict the metals cations from interfering with the cells’ biochemical pathways and cell signaling (Thumann et al., 1991; Maitani et al., 1996).

#### 5.4.5 Compartmentalization/Detoxification of heavy metals

The plants either transport a heavy metal out of the cell or restrict it to the vacuole and then detoxification occurs once a heavy metal enters the cytosol, a process called sequestration/detoxification (Kanoun-Boulé et al., 2009; Singh et al., 2011). Thus, heavy metals are restricted from interference with vital metabolic pathways. This process allows plants to survive under metal-contaminated areas without toxic effect. Several transporter families are involved in this process, which includes ABC, CDF, HMA, and NRAMP transporters. Several studies show high concentrations of Cd and Zn in the vacuole of a cell. For instance, the nickel hyperaccumulator *Alyssum serpytllifolium* gathered up to 72% of Ni in the vacuole (Brooks et al., 1980; Ernst et al., 1992).

## 6 Conclusion

Heavy metals contamination is one of the greatest threats to human health and the survival of other living organisms, including plants. This threat increases with increasing industrialization in developed and developing countries. Heavy metals are non-biodegradable, which remain in the environment. Decontaminating soils and water bodies from heavy metal contamination is an economically expansive process. Thus, recent research is focused on finding plants for the phytoremediation of heavy metals. However, most of the plants are prone to the negative effects of heavy metals toxicity, which affect their growth and yield and have far-ranging impacts on various aspects of these plants. Some plants employ certain strategies to cope with heavy metal toxicity. These strategies may include anatomical changes within the plant organs, such as thickening cell walls to inhibit heavy metals into the cells or physiological adaptations, such as sequestration or molecular responses. such as chelation. All these responses depend on cell signaling within the plants. The cell signaling pathways adjust according to the concentration and the type of heavy metal pollution.

## 7 Future prospects

It is recommended that the researchers should study some model plants, including their plant morphology, anatomy, physiology, molecular biology, cell signaling, and genetics under heavy metal stress. It will further answer how heavy metals trigger certain signaling pathways and how those signals are translated into morphological, anatomical, physiological, and biochemical responses. It is also necessary to recognize the genes that are accountable for controlling all of these processes. The factors that restrict or facilitate the uptake, translocation, and sequestration of heavy metal ions in plants and that have been genetically modified to have high biomass and rapid growth rate should be the focus of future research. This will enable the practical application of knowledge in forming transgenic types, which are more effective at phytoremediation and have better capacities for tolerance against the toxic heavy metals. We now have a better understanding of stress tolerant mechanisms with the development of novel omics technologies for cellular complexity research. Numerous stress-related genes have already been discovered with this advanced sequencing technologies. Unexpected outcomes have been attained by genetically modifying metabolites, proteins, and heavy metal stress responsive genes. The full potential of phenomics and functional genomics must be utilized.

It may be possible to lessen the load of heavy metals on agriculture by using nano-particles for the adsorption and co-adsorption of heavy metal ions from irrigation water. Another crucial area that could aid in achieving environmental sustainability is using bio-indicator plants to monitor heavy metal hot spots. Further research investment is required to understand better the interactions between plants and microbes under heavy metal stress, as this information may help develop practical strategies for recovering soils polluted with heavy metals.

## 8 Limitations

This review focuses on various genetic, molecular, and cell signaling levels that work together to produce a coordinated response to heavy metal toxicity and deduce the mechanisms behind the tolerance. However, many important questions still need to be clarified because not all heavy metals cause the same physiological and biochemical reactions in plants. Similarly, how different plants react differently to various heavy metals. Because of these, it is challenging to determine a single stress-induced pathway that protects plants from all heavy metals. Good basic knowledge of the antioxidative mechanisms in plants is needed for much of the in-depth study.

## Author contributions

UE: Conceptualization; Formal analysis; Investigation; Literature review; Validation; Visualization; Roles/Writing - original draft; SK and AR: Conceptualization; Formal analysis; Investigation; Project administration; Resources; Supervision; Validation; Visualization; Writing - review and editing; NK: Visualization; Writing - review and editing; Validation; ZA and SJ: Formal analysis; Investigation; Methodology; Validation; Visualization; ZF: Writing - review and editing; LL and HH: Writing - review and editing; Funding acquisition. All authors contributed to the article and approved the submitted version.

## In Memoriam

We dedicate this article to our teacher and an incredible Plant Scientist Prof Dr Habib Ahmad (TI), who left this mortal world on the night of 6-7th April 2021 due to COVID 19; while this paper has been accepted on 6-7th April 2023 on the occasion of his death anniversary.
